# Changes in the 3D Corneal Structure and Morphogeometric Properties in Keratoconus after Corneal Collagen Crosslinking

**DOI:** 10.3390/diagnostics10060397

**Published:** 2020-06-11

**Authors:** Ramón Alifa, David Piñero, José Velázquez, Jorge L. Alió del Barrio, Francisco Cavas, Jorge L. Alió

**Affiliations:** 1 Doctorate Program in Industrial Technologies, International School of Doctorate, Technical University of Cartagena, 30202 Cartagena, Spain; ramon.alifa@gmail.com; 2 Group of Optics and Visual Perception, Department of Optics, Pharmacology and Anatomy, University of Alicante, 03690 Alicante, Spain; david.pinyero@gcloud.ua.es; 3 Department of Structures, Construction and Graphical Expression, Technical University of Cartagena, 30202 Cartagena, Spain; jose.velazquez@upct.es; 4 Department of Research and Development, VISSUM, 03016 Alicante, Spain; jorge_alio@hotmail.com (J.L.A.d.B.); jlalio@vissum.com (J.L.A.); 5 Cornea, Cataract and Refractive Surgery Department, VISSUM, 03016 Alicante, Spain; 6 Division of Ophthalmology, Department of Pathology and Surgery, Faculty of Medicine, Miguel Hernández University, 03202 Alicante, Spain

**Keywords:** anterior segment, tomography, surgery, geometry, CXL

## Abstract

Keratoconus is an ectatic disorder that is presently considered one of the most prevalent reasons for keratoplasty. Corneal collagen crosslinking (CXL) is the only proven treatment option available that is capable of halting the progression of the disease by stabilizing the cone in 90% of cases, and by also reducing refractive error and maximal keratometry. This study assesses, by means of a 3D morphogeometric analysis procedure developed by our research team, the corneal structure changes that occur immediately after CXL treatment and during a 6 month follow-up period. A total of 19 eyes from 19 patients diagnosed with keratoconus who underwent CXL were included, and several variables derived from the morphogeometric analysis were calculated and evaluated for the pre-operative, 3 month postoperative, and 6 month postoperative states. Significant reductions were detected in central corneal thickness and corneal spherical-like root mean square (RMS) 3 months after surgery, with non-significant regression of the effect afterward. Significant reductions in the total corneal area/volume were found, with some levels of regression after 6 months in certain volumetric parameters. In conclusion, the eyes with higher values for morphogeometric parameters—posterior apex deviation (PAD), anterior minimum thickness point deviation (AMTPD), and posterior minimum thickness point deviation (PMTPD)—seemed more likely to undergo aberrometric improvement as a result of CXL surgery.

## 1. Introduction

Keratoconus (KC) is an ectatic disorder characterized by progressive thinning, steepening, and distortion of the cornea, with secondary loss of vision due to unacceptable levels of irregular astigmatism [1]. If left untreated, about 12–20% of keratoconus patients will require corneal transplantation for their visual rehabilitation [2], which implies several potential drawbacks, such as graft rejection, failure, and long visual recovery [1]. In actuality, keratoconus is one of the most prevalent reasons for keratoplasty [3].

Corneal collagen crosslinking (CXL) is the only available treatment option that has been demonstrated to halt the progression of the disease [4]. CXL, first introduced by Spoerl and Seiler at the University of Dresden in 1996 [5], consists in inducing the formation of chemical bridges (crossed links) between the collagen fibers within the corneal stroma to increase their stiffness and to thus avoid further cornea deformation. This is achieved by irradiation with ultraviolet A light (UVA), performed on a corneal stroma previously soaked with riboflavin (vitamin B2). CXL not only stabilizes the cone at a success rate over 90%, but also improves ectasia to some extent with an average reduction of 1D in refractive error, and of 2D in maximal keratometry, while visual acuity remains unchanged or improves by about 1–2 lines [6].

Advances in diagnostic technologies allow a more comprehensive analysis of the corneal structure, which is not limited only to the geometry of the anterior and posterior corneal surfaces [7]. This may be especially useful for understanding the changes that occur in the cornea after CXL. Our research group developed a new method to perform a more complete morphogeometric analysis of the cornea on the basis of the data obtained from a Scheimpflug imaging-based tomography system [8]. This advanced corneal analysis has been demonstrated to be useful for characterizing the corneal structure in patients with keratoconus [9,10] for subclinical and clinical keratoconus detection [11,12,13], patient education [14], and even for characterizing the symmetry of the corneal profile between both eyes of healthy patients [15]. The aim of the current study was to conduct this 3D morphogeometric analysis to evaluate corneal changes after CXL in keratoconus patients and during a 6 month follow-up period.

## 2. Materials and Methods

This cross-sectional research followed Declaration of Helsinki guidelines for the use of human subjects, and was endorsed by the Institutional Ethical Board of the VISSUM Alicante clinic.

### 2.1. Study Population

This research included 19 eyes from 19 patients (ages ranging from 18 to 69) diagnosed with KC and undergoing CXL. They were all included in the IBERIA database for keratoconus, and were obtained at the VISSUM Innovation, Cornea, Cataract and Refractive Surgery Unit, Alicante, Spain, which is a center affiliated with the Miguel Hernandez University of Elche (Alicante, Spain).

A single experienced ophthalmologist verified the KC diagnosis by checking the presence of the following signs: evidence for KC in retinoscopy and bio-microscopy (e.g., Vogt striae, Munson sign, Fleischer ring, Rizzuti phenomenon, scissoring), existence of topographical patterns associated with KC on the axial curvature map (round, oval, irregular, inferior step with/without skewed radial axes (SRAX) over 21 degrees, asymmetric bowtie, and 3 mm inferior–superior mean keratometric difference bigger than 1.4 D), central/paracentral or inferior focal steepening (anterior and/or posterior), and/or corneal thickness reduction.

### 2.2. Surgical Procedure

All the surgical procedures were performed by the same experienced surgeon under sterile conditions in an operating room. Before surgery, topical and peribulbar anesthesias were applied and an eyelid speculum was inserted to avoid patients blinking during the surgical procedure and to isolate the corneal surgical zone. The corneal epithelium was mechanically debrided with a manual scraper over an entire 9 mm area from the center of the cornea. A Dresden CXL protocol was applied as previously described [5]. Briefly, isotonic riboflavin was topically applied to the cornea every 2 min for 30 min (by intraoperative pachymetric monitoring with ultrasound to ensure a minimal corneal thickness of 400 microns), followed by 3 mW/cm UVA radiation for 30 min. A topical combination of antibiotic and dexamethasone (Tobradex, Alcon Cusi, Barcelona, Spain), together with a soft bandage contact lens, were applied at the end of the procedure. The bandage contact lens was kept until full re-epithelialization.

The following were prescribed to be applied: topical moxifloxacin hydrochloride drops (Vigamox, Alcon Cusi, Barcelona, Spain), 4 times daily for 7 days; dexamethasone 0.1% drops (Maxidex, Alcon Cusi, Barcelona, Spain), 4 times daily in a tapering manner for a total 4 week period; 0.3% hydroxypropyl methylcellulose drops (Tears Naturale; Alcon Cusi, Barcelona, Spain), 6 times daily for 2 months.

### 2.3. Examinations and Measurements

Thorough ophthalmological examinations were made on all subjects, such as retinoscopy, corrected distance visual acuity (CDVA) assessment, Goldman’s tonometry, slit-lamp bio-microscopy, and dilated fundus examination. A single experienced technician took three consecutive topographical measurements with a Sirius System (CSO, Florence, Italy). Only the measures showing green-colored checkmarks, which correspond to best acquisition quality, were selected for the study. Hereafter, the points of clouds representing both the anterior and posterior corneal topographies were exported in the. CSV format to be subsequently studied in detail by the morphogeometric analysis procedure established and validated by our research team [8]. The same examination protocol was conducted at 3 and 6 months post-surgery.

### 2.4. Morphogeometric Analysis

**Figure 1:**
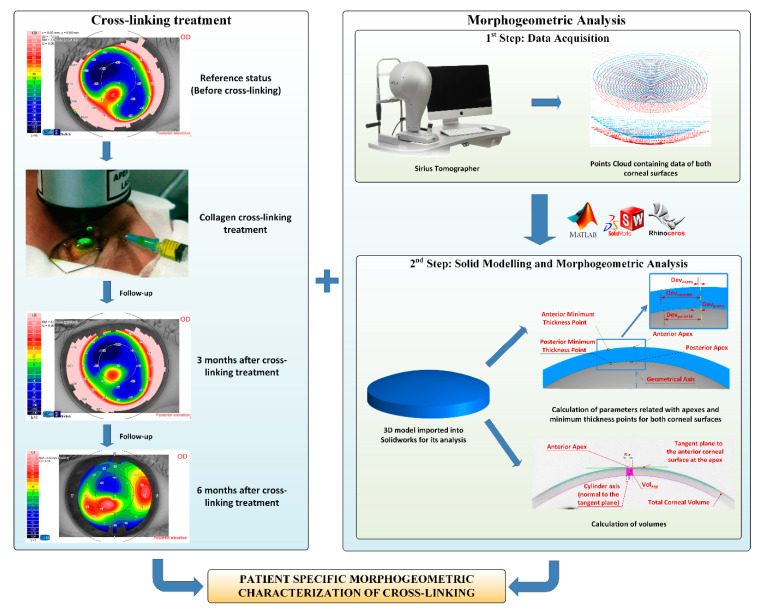
Sequence in the cross-linking treatment, and its follow-up (left) and procedure for the 3D corneal model generation and later analysis (right). The data obtained from the Sirius tomographer permitted the generation of a customized 3D model, in which several morphogeometric variables were studied.

The morphogeometric analysis procedure applied in this study consisted of two consecutive steps (Figure 1).

- Data Acquisition. A custom-made script programmed in the Matlab R2017b (Mathworks, Natick, USA) software was created to transform each point of the cloud provided by the tomographer in polar coordinates into the Cartesian format. This procedure has been meticulously described in several previous research works [8,9,10,11,12,13,14,15]. The program provides a. CSV file containing data of the points of clouds that describe both the anterior and posterior corneal surfaces for the area comprised between the geometric center of the cornea (r = 0 mm) and the mid-peripheral area (r = 4 mm). This zone usually contains most corneal anomalies for not only diseased, but also healthy eyes [8,9,10,11,12,13,14,15]. Afterward, both surfaces were imported to the Rhinoceros V 5.0 (MCNeel and Associates, Seattle, USA) software, and the “patch” function was used to find the surfaces that better fitted the clouds of points. The fixed configuration settings for the function were:
  • Sample point spacing: 256;
  • Surface span planes: 255 for both directions “u” and “v”;
  • Stiffness of the solution surface: 1.
- Solid modeling and morphogeometric analysis. Finally, a customized three-dimensional model of the patient’s cornea was created from the surfaces previously generated in Rhinoceros by importing them to the SolidWorks V2018 (Dassault Systèmes, Vélizy-Villacoublay, France) software [8,9,10,11,12,13,14,15]. This model was then analyzed, and several morphogeometrical parameters were established. These parameters herein studied, as well as their particularities, have been previously described in several other articles [8,9,10,11,12,13,14,15]. This was also the case of several volumetric parameters related directly to the volumes around the anterior/posterior apices and the minimum thickness points [12].

**Figure 2:**
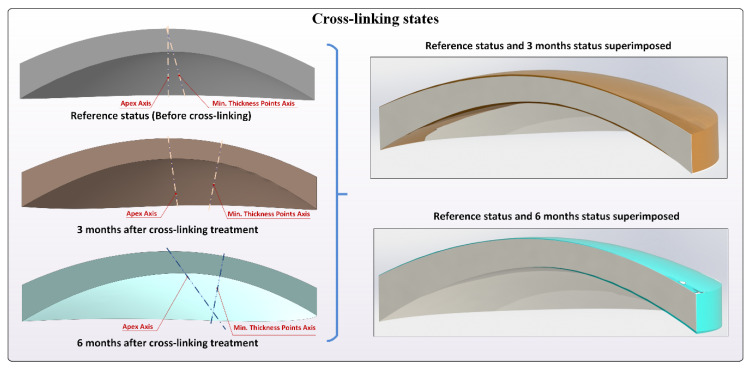
Different crosslinking states, preoperation, 3 months postoperative, and 6 months postoperative. Lines joining apex points and minimum thickness points of both corneal surfaces are included.

In conclusion, the following variables derived from the above-described morphogeometric analysis were calculated and evaluated for the pre-operative, 3 month postoperative, and 6 month postoperative states (Figure 2) [8,9,10,11,12,13,14,15]:

- A_ant_: area of the anterior corneal surface [9].
- A_post_: area of the posterior corneal surface [9].
- A_tot_: total area of the corneal surface [9].
- CV: total cornea volume [9].
- AAD: anterior apex deviation, which is the minimum mean distance between the point of maximum curvature (apex) of the anterior corneal surface and the *z*-axis [11].
- PAD: posterior apex deviation, which is the minimum mean distance between the point of maximum curvature of the posterior corneal surface and the *z*-axis [11].
- AMTPD: anterior minimum thickness point deviation, which is the minimum distance between the anterior minimum thickness point and the optical axis that goes through the geometrical center of the anterior corneal surface [9,12].
- PMTPD: posterior minimum thickness point deviation, which is the minimum distance between the posterior minimum thickness point and the optical axis that goes through the geometrical center of the anterior corneal surface [9,12].
- VOLmct: volume contained in the intersection between the solid model of the cornea and a cylinder of revolution with radius × (from 0.1 to 1.5 mm) and its axis defined by the points of minimum corneal thickness of the anterior and posterior corneal surface [9,12].
- VOLaap: volume contained in the intersection between the solid model of the cornea and a cylinder of revolution with radius × (from 0.1 to 1.5 mm) and its axis defined by a straight line perpendicular to the tangent plane to the anterior corneal surface at the apex [9,12].
- VOLpap: volume contained in the intersection between the solid model of the cornea and a cylinder of revolution with radius × (from 0.1 to 1.5 mm) and its axis defined by a straight line perpendicular to the tangent plane to the posterior corneal surface at the apex [9,12].

### 2.5. Statistical Analysis

Statistical analyses were performed with a commercially available software package (SPSS for Mac, Version 20.0; IBM Corporation, Armonk, NY, USA). Data variables were confirmed as being non-normally distributed by means of the Kolmogorov–Smirnov test. The Friedman test was used to assess the statistical significance of differences between consecutive visits (preoperative and postoperative). The Wilcoxon tests with Bonferroni correction were used to analyze the differences between pairs of visits. The Spearman correlation coefficient was utilized to assess the level of correlation between the changes in clinical variables at 3 months post-surgery and the different preoperative data. For all the statistical tests, a *p*-value of less than 0.05 was considered to be statistically significant.

## 3. Results

The evaluated dataset included a total of 19 eyes (7 right and 12 left eyes) of 19 patients (13 males and 6 females) who underwent CXL surgery with 6 month postoperative follow-up. Table 1 summarizes the changes that occurred in the evaluated different clinical variables. As shown, significant reductions were detected during the follow-up in the central corneal thickness (CCT) (*p* = 0.006) and the corneal spherical-like root mean square (RMS) (*p* = 0.030) at 3 months after surgery, with a non-significant regression of the effect afterward (*p* = 0.084). Furthermore, a change in the limit of statistical significance was found in the coma-like RMS during follow-up (*p* = 0.057). Likewise, reductions were observed in manifest sphere, cylinder, spherical equivalent (SE), mean keratometry, and total and primary coma RMS (*p* ≥ 0.115). 

Table 2 summarizes the changes in the corneal morphogeometric parameters defined during the follow-up in the analyzed sample. As shown, a statistically significant reduction was observed in A_tot_ (*p* = 0.001) and CV (*p* < 0.001), with no significant changes afterward (*p* ≥ 0.816). No significant changes were detected during the follow-up for the other calculated and evaluated morphogeometric parameters (*p* ≥ 0.076). The change in total CV after 3 months showed a moderate but statistically significant correlation with the change in CCT (*r* = 0.594, *p* = 0.042), and also with preoperative corneal asphericity in the 4.5 mm central area (*r* = 0.503, *p* = 0.047) and spherical aberration (*r* = 0.577, *p* = 0.019). However, the change in CV at 3 months did not correlate with the changes in the spherical-like (*r* = 0.063, *p* = 0.846) and coma-like RMS (*r* = 0.056, *p* = 0.863). In contrast, the change in the spherical-like RMS at 3 months after surgery correlated significantly with preoperative PAD (*r* = −0.699, *p* = 0.011), while the change in the coma-like RMS correlated significantly with the preoperative coma-like RMS (*r* = −0.688, *p* = 0.013), AMTPD (*r* = −0.671, *p* = 0.017), PMTPD (*r* = −0.692, *p* = 0.013), and spherical equivalent (*r* = 0.577, *p* = 0.049). Furthermore, a significant correlation was also noted between the change in CCT and AMTPD (*r* = −0.692, *p* = 0.013).

Table 3 summarizes the post-surgery volumetric changes during follow-up in the analyzed sample. As shown, statistically significant reductions in all the defined corneal volume parameters (VOL_mct_, VOL_aap_, and VOL_pap_ for a radius from 0.1 mm to 1.5 mm) were found 3 months after surgery (*p* ≤ 0.002), with some level of regression at the end of the follow-up.

## 4. Discussion

Several studies have shown that some microstructural changes occur after CXL in eyes with keratoconus [16,17,18,19,20]. These microstructural changes allow the anterior corneal stroma (300 µm) to stiffen after surgery, which is related to a crosslinking of collagen fibers [21]. Specifically, UVA CXL leads to changes in the collagen fibril network of the cornea due to stromal edema and interfibrillar spacing narrowing [17]. CXL induces interlacing lamellae in the anterior stroma, followed by well-organized parallel running lamellae containing uniformly distributed collagen fibrils decorated with normal proteoglycans [16]. It has also been demonstrated that the diameter of collagen fibrils and interfibrillar spacing after CXL significantly increase compared to those in untreated keratoconus corneas, whereas the proteoglycan area is significantly smaller [16]. Collagen crimping is also reduced by approximately 1%, possibly due to the shortening of the collagen fibers over the crosslinked region of the cornea [20]. All these modifications are associated with a complete loss of the sub-basal nerve plexus and anterior stromal keratocytes during the early postoperative period, with near-complete regeneration by 12 months postoperatively [18]. In addition, these microstructural modifications are associated with some clinical changes that can be detected by the diagnostic and imaging technologies currently available in clinical settings, including mainly corneal thinning, reduction of corneal volume, and improvement in some corneal topographic indices [22,23,24,25,26,27,28,29,30,31]. The aim of the current study was to evaluate the changes that occur in the corneal structure by an advanced morphogeometric analysis [8,12] after CXL in a sample of keratoconus eyes during a 6 month follow-up.

In the current series, a significant pachymetric reduction was observed at 3 months post-surgery, with a mean change of 34.5 µm, and a non-significant minimal regression of the effect afterward. This is consistent with previous studies on CXL outcomes showing significant corneal thinning during the early postoperative period, which is partially maintained during the follow-up [22,23,27,29,31]. This central corneal thinning was consistent with the significant reduction of CV (mean change: 0.99 mm^3^), with a significant positive correlation between the changes in CV and CCT. Other authors have also reported a significant reduction in corneal volume after CXL surgery as calculated by different approaches [22,23,25,27,29]. Besides calculating herein the total corneal volume, we also calculated the volumes contained in the intersection between the solid model of the generated cornea, as well as a cylinder of revolution with an increasing radius from 0.1 to 1.5 mm and axis defined according to different anatomical reference points. All these volumes significantly reduced after CXL, independently of the reference point used to define the axis of the cylinder of revolution. This means that the reduction in corneal volume was global, and it affected the whole area to which CXL treatment was applied. More studies are needed to corroborate the effect of CXL on selective cornea areas, such as its apex or a thinner area.

For the defined morphogeometric parameters, we found significant changes with surgery only in A_tot_, which is coherent if we consider the reduction in corneal thickness and CV. This confirms that the reduction in corneal volume in keratoconus with CXL may have different geometric distribution effects for both corneal surfaces. Indeed the variability among patients in the change observed in parameters such as PAD, AMTPD, or PMTPD was wide. However, the level of irregularity of the corneal structure defined preoperatively by morphogeometric parameters correlated significantly with the changes recorded 3 months after surgery in spherical-like and coma-like aberrations. Specifically, negative correlations were found between the changes in both spherical-like RMS and preoperative PAD, and also in the changes in the coma-like RMS and preoperative AMTPD and PMTPD. This means that a more aberrometric reduction was achieved in those keratoconus cases with higher PAD, AMTPD, and PMTPD values, which is consistent with more severe stages of this disease [13]. This suggests that the keratoconus eyes with more relevant alterations to their structure are more susceptible to improvements in aberrometric terms in relation to the effect of CXL. This is a finding that should be investigated in future trials.

In conclusion, CXL induces a significant thinning and decrease in the total and local corneal volume of keratoconus eyes, which relatively continued during the 6 month follow-up. The eyes with higher PAD, AMTPD, and PMTPD values seemed more susceptible to undergoing aberrometric improvement after CXL surgery. The morphogeometric analysis developed by our research group proved to be a useful tool for evaluating and monitoring post-CXL changes in ectatic corneas.

## Figures and Tables

**Table 1:** Visual, refractive, corneal topographic, and aberrometric outcomes during the follow-up in the analyzed sample. Abbreviations: SD, standard deviation; D, diopters; UDVA, uncorrected distance visual acuity; SE, spherical equivalent; CDVA, corrected distance visual acuity; CCT, central corneal thickness; KM, mean keratometry; Q_4.5_, asphericity in the central 4.5 mm corneal area; Q_8_, asphericity in the central 8.0 mm corneal area; RMS, root mean square.

Mean (SD) Median (Range)	Preoperative	3 Months Postop	6 Months Postop	*p*-Value
LogMAR UDVA	0.66 (0.58) 0.52 (0.01 to 2.00)	0.56 (0.51) 0.40 (0.10 to 2.00)	0.66 (0.68) 0.38 (0.00 to 2.00)	0.449
Manifest sphere (D)	−0.75 (2.48) 0.00 (−6.25 to 3.75)	−0.40 (2.57) 0.00 (−6.50 to 3.25)	−1.41 (2.80) −0.75 (−6.50 to 1.25)	0.949
Manifest cylinder (D)	−2.97 (2.00) −2.25 (−9.00 to −0.50)	−2.63 (1.35) −2.50 (−4.75 to 0.00)	−2.59 (1.06) −3.00 (−4.75 to −1.00)	0.465
Manifest SE (D)	−2.24 (2.47) −2.25 (−7.00 to 2.88)	−1.72 (2.48) −1.38 (−7.50 to 2.50)	−2.70 (2.61) −2.13 (−7.50 to 0.75)	0.538
LogMAR CDVA	0.09 (0.09) 0.09 (−0.04 to 0.30)	0.12 (0.11) 0.10 (0.00 to 0.32)	0.10 (0.11) 0.02 (0.00 to 0.28)	0.438
CCT (µm)	496.42 (34.99) 495.00 (444 to 564)	461.92 (36.82) 463.00 (414 to 520)	468.73 (46.69) 484.00 (377 to 531)	0.006 Preop—3 months 0.006 3–6 months 0.084 Preop—6 months 0.024
KM (D)	45.44 (2.00) 44.88 (42.16 to 48.82)	44.36 (3.01) 43.51 (39.49 to 49.26)	45.47 (2.80) 45.62 (41.40 to 50.27)	0.311
Q_45_	−0.58 (0.79) −0.39 (−2.00 to 0.51)	−0.96 (0.84) −1.08 (−2.17 to 0.36)	−0.81 (1.17) −0.40 (−2.91 to 1.12)	0.846
Q_8_	−0.67 (0.32) −0.68 (−1.35 to −0.16)	−0.69 (0.47) −0.81 (−1.13 to 0.51)	−0.63 (0.38) −0.51 (−1.37 to −0.17)	0.115
Total RMS (µm)	4.92 (3.73) 4.13 (1.07 to 14.39)	3.65 (1.48) 4.09 (1.25 to 6.20)	4.45 (3.27) 4.33 (1.04 to 13.65)	0.115
Astigmatism RMS (µm)	2.74 (1.14) 2.91 (0.78 to 4.53)	2.69 (1.17) 2.47 (0.73 to 4.14)	2.68 (1.34) 3.04 (0.78 to 4.88)	0.135
Primary spherical aberration (µm)	0.02 (0.36) 0.04 (−0.53 to 0.56)	−0.10 (0.30) −0.06 (−0.61 to 0.36)	0.15 (0.37) 0.12 (−0.52 to 0.78)	0.311
Primary coma RMS (µm)	1.64 (0.97) 1.49 (0.26 to 3.71)	1.21 (0.95) 0.98 (0.04 to 3.05)	1.53 (1.04) 1.45 (0.12 to 3.33)	0.223
Spherical-like RMS (µm)	0.75 (0.36) 0.64 (0.23 to 1.51)	0.67 (0.36) 0.57 (0.27 to 1.36)	0.84 (0.47) 0.77 (0.18 to 1.65)	0.030 Preop—3 months 0.015 3–6 months 0.084 Preop—6 months 0.722
Coma-like RMS (µm)	1.85 (1.00) 1.60 (0.38 to 3.98)	1.65 (0.86) 1.41 (0.43 to 3.19)	1.86 (0.88) 1.87 (0.59 to 3.05)	0.057

**Table 2:** Changes in the corneal morphogeometric parameters defined during the follow-up in the analyzed sample. Abbreviations: SD, standard deviation; A_ant_, anterior corneal surface area; A_post_, posterior corneal surface area; A_tot_, total corneal surface area; CV, corneal volume; AAD, anterior apex deviation which is the average distance from the *z*-axis to the highest point (apex) of the anterior corneal surface; PAD, posterior apex deviation which is the average distance from the *z*-axis to the highest point (apex) of the posterior corneal surface; AMTPD, anterior minimum thickness point deviation which is the average distance on the *xy*-plane from the *z*-axis to the minimum thickness point of the anterior corneal surface; PMTPD, posterior minimum thickness point deviation which is the average distance on the *xy*-plane from the *z*-axis to the minimum thickness point of the anterior corneal surface.

Mean (SD) Median (Range)	Preoperative	3 Months Postop	6 Months Postop	*p*-Value
A_ant_ (mm^2^)	43.27 (0.26) 43.22 (42.93 to 43.81)	43.16 (0.27) 43.08 (42.85 to 43.79)	43.24 (0.32) 43.15 (42.83 to 43.75)	0.205
A_post_ (mm^2^)	44.60 (0.51) 44.41 (43.94 to 45.64)	44.56 (0.57) 44.30 (44.00 to 45.83)	44.72 (0.48) 44.49 (44.24 to 45.71)	0.338
A_tot_ (mm^2^)	104.07 (2.00) 103.50 (100.97 to 107.68)	102.98 (2.26) 102.59 (100.30 to 106.79)	103.87 (1.81) 102.95 (101.37 to 106.77)	0.001 Preop—3 months 0.006 3–6 months 0.999 Preop—6 months 0.003
CV (mm^3^)	24.61 (1.95) 24.16 (21.38 to 27.97)	22.65 (1.91) 22.81 (20.37 to 26.32)	23.62 (1.89) 23.28 (19.70 to 26.87)	<0.001 Preop—3 months <0.001 3–6 months 0.816 Preop—6 months 0.003
AAD (mm)	0.0098 (0.0091) 0.0080 (0.0000 to 0.0400)	0.0084 (0.0066) 0.0068 (0.0000 to 0.0200)	0.0113 (0.0139) 0.0061 (0.000 to 0.0500)	0.779
PAD (mm)	0.21 (0.09) 0.17 (0.10 to 0.39)	0.23 (0.11) 0.20 (0.10 to 0.51)	0.26 (0.13) 0.28 (0.09 to 0.45)	0.076
AMTPD (mm)	1.04 (0.30) 1.03 (0.60 to 1.75)	1.14 (0.39) 1.22 (0.30 to 1.93)	1.28 (0.41) 1.29 (0.66 to 2.18)	0.717
PMTPD (mm)	0.97 (0.27) 0.94 (0.58 to 1.62)	1.08 (0.37) 1.16 (0.26 to 1.86)	1.19 (0.41) 1.20 (0.60 to 2.08)	0.717

**Table 3:** Changes in the corneal volumetric parameters defined during the follow-up in the analyzed sample. Abbreviations: SD, standard deviation; VOL_mct_, volume contained in the intersection between the solid model of the cornea and a cylinder of revolution with radius × (from 0.1 to 1.5 mm) and its axis defined by the points of the minimum corneal thickness of the anterior and posterior corneal surface; VOL_aap_, volume contained in the intersection between the solid model of the cornea and a cylinder of revolution with radius × (from 0.1 to 1.5 mm) and its axis defined by a straight line perpendicular to the tangent plane to the anterior corneal surface at the apex; VOL_pap_, volume contained in the intersection between the solid model of the cornea and a cylinder of revolution with radius × (from 0.1 to 1.5 mm) and its axis defined by a straight line perpendicular to the tangent plane to the posterior corneal surface at the apex.

Mean (SD) Median (Range)	Preoperative	3 Months Postop	6 Months Postop	*p*-Value
VOLmct (mm^3^)				
Radius				
0.1 mm	0.015 (0.001) 0.015 (0.014–0.017)	0.013 (0.001) 0.013 (0.011–0.015)	0.014 (0.002) 0.014 (0.010–0.016)	0.002
0.2 mm	0.060 (0.004) 0.060 (0.054–0.069)	0.054 (0.005) 0.053 (0.043–0.060)	0.054 (0.007) 0.056 (0.042–0.063)	0.002
0.3 mm	0.134 (0.087) 0.134 (0.122–0.156)	0.121 (0.012) 0.120 (0.098–0.136)	0.122 (0.015) 0.126 (0.095–0.142)	0.002
0.4 mm	0.241 (0.016) 0.239 (0.217–0.279)	0.216 (0.021) 0.214 (0.176–0.243)	0.219 (0.026) 0.225 (0.170–0.253)	0.002
0.5 mm	0.377 (0.024) 0.374 (0.340–0.437)	0.335 (0.034) 0.323 (0.277–0.381)	0.344 (0.040) 0.353 (0.268–0.397)	0.002
0.6 mm	0.545 (0.035) 0.541 (0.491–0.631)	0.490 (0.047) 0.485 (0.403–0.551)	0.497 (0.057) 0.510 (0.391–0.574)	0.002
0.7 mm	0.745 (0.049) 0.738 (0.670–0.862)	0.670 (0.064) 0.664 (0.555–0.753)	0.680 (0.078) 0.697 (0.539–0.784)	0.002
0.8 mm	0.977 (0.064) 0.968 (0.877–1.130)	0.880 (0.083) 0.871 (0.734–0.988)	0.894 (0.100) 0.914 (0.709–1.030)	0.002
0.9 mm	1.243 (0.082) 1.230 (1.112–1.435)	1.121 (0.104) 1.109 (0.941–1.256)	1.139 (0.126) 1.162 (0.905–1.310)	0.002
1.0 mm	1.542 (0.103) 1.526 (1.378–1.779)	1.392 (0.126) 1.376 (1.179–1.557)	1.416 (0.154) 1.441 (1.128–1.626)	0.002
1.1 mm	1.877 (0.127) 1.855 (1.675–2.163)	1.694 (0.151) 1.675 (1.449–1.891)	1.725 (0.185) 1.753 (1.378–1.979)	0.002
1.2 mm	2.248 (0.153) 2.221 (2.000–2.588)	2.030 (0.178) 2.004 (1.750–2.265)	2.070 (0.218) 2.097 (1.653–2.371)	0.002
1.3 mm	2.655 (0.183) 2.623 (2.357–3.052)	2.400 (0.206) 2.371 (2.085–2.685)	2.449 (0.254) 2.477 (1.961–2.802)	0.001
1.4 mm	3.100 (0.215) 3.063 (2.747–3.558)	2.803 (0.237) 2.769 (2.458–3.145)	2.863 (0.292) 2.900 (2.297–3.267)	<0.001
1.5 mm	3.585 (0.251) 3.542 (3.168–4.109)	3.241 (0.269) 3.204 (2.873–3.647)	3.317 (0.331) 3.369 (2.668–3.778)	<0.001
VOLaap (mm^3^)				
Radius				
0.1 mm	0.016 (0.001) 0.016 (0.014–0.018)	0.014 (0.001) 0.014 (0.012–0.016)	0.014 (0.004) 0.015 (0.000–0.017)	<0.001
0.2 mm	0.062 (0.004) 0.062 (0.056–0.071)	0.057 (0.005) 0.057 (0.047–0.064)	0.059 (0.006) 0.060 (0.047–0.067)	<0.001
0.3 mm	0.140 (0.010) 0.140 (0.125–0.160)	0.128 (0.011) 0.129 (0.107–0.144)	0.132 (0.014) 0.135 (0.105–0.150)	<0.001
0.4 mm	0.250 (0.017) 0.249 (0.223–0.284)	0.228 (0.019) 0.229 (0.191–0.256)	0.234 (0.024) 0.240 (0.188–0.268)	<0.001
0.5 mm	0.391 (0.027) 0.390 (0.349–0.445)	0.356 (0.030) 0.358 (0.300–0.401)	0.367 (0.037) 0.375 (0.296–0.419)	<0.001
0.6 mm	0.565 (0.039) 0.562 (0.503–0.643)	0.514 (0.042) 0.515 (0.435–0.578)	0.530 (0.053) 0.541 (0.430–0.604)	<0.001
0.7 mm	0.770 (0.053) 0.767 (0.685–0.877)	0.701 (0.057) 0.700 (0.597–0.789)	0.722 (0.073) 0.737 (0.589–0.825)	<0.001
0.8 mm	1.008 (0.069) 1.004 (0.897–1.149)	0.917 (0.074) 0.914 (0.786–1.033)	0.946 (0.094) 0.965 (0.774–1.080)	<0.001
0.9 mm	1.280 (0.088) 1.274 (1.137–1.459)	1.164 (0.093) 1.157 (1.005–1.312)	1.201 (0.119) 1.222 (0.982–1.371)	<0.001
1.0 mm	1.585 (0.109) 1.578 (1.406–1.807)	1.441 (0.114) 1.430 (1.253–1.624)	1.488 (0.147) 1.512 (1.215–1.699)	<0.001
1.1 mm	1.925 (0.133) 1.916 (1.705–2.195)	1.750 (0.137) 1.732 (1.533–1.974)	1.807 (0.177) 1.834 (1.476–2.066)	<0.001
1.2 mm	2.300 (0.159) 2.288 (2.034–2.624)	2.089 (0.163) 2.062 (1.842–2.362)	2.158 (0.211) 2.187 (1.759–2.466)	<0.001
1.3 mm	2.710 (0.188) 2.695 (2.394–3.094)	2.461 (0.190) 2.424 (2.184–2.784)	2.544 (0.247) 2.574 (2.071–2.906)	<0.001
1.4 mm	3.156 (0.220) 3.140 (2.784–3.604)	2.867 (0.218) 2.821 (2.562–3.242)	2.965 (0.286) 2.996 (2.407–3.392)	<0.001
1.5 mm	3.642 (0.255) 3.621 (3.206–4.158)	3.308 (0.248) 3.251 (2.984–3.743)	3.422 (0.328) 3.453 (2.779–3.922)	0.001
VOLpap (mm^3^)				
Radius				
0.1 mm	0.015 (0.001) 0.015 (0.014–0.018)	0.014 (0.001) 0.014 (0.011–0.016)	0.014 (0.015) 0.014 (0.011–0.016)	<0.001
0.2 mm	0.062 (0.004) 0.062 (0.056–0.070)	0.056 (0.005) 0.056 (0.045–0.062)	0.057 (0.006) 0.058 (0.044–0.065)	<0.001
0.3 mm	0.139 (0.009) 0.140 (0.125–0.159)	0.125 (0.011) 0.125 (0.102–0.140)	0.129 (0.014) 0.131 (0.099–1.467)	<0.001
0.4 mm	0.247 (0.016) 0.248 (0.222–0.282)	0.223 (0.020) 0.221 (0.182–0.249)	0.229 (0.024) 0.233 (0.178–0.261)	<0.001
0.5 mm	0.386 (0.025) 0.389 (0.348–0.442)	0.350 (0.031) 0.346 (0.286–0.389)	0.359 (0.037) 0.365 (0.280–0.409)	0.002
0.6 mm	0.557 (0.037) 0.561 (0.501–0.638)	0.505 (0.045) 0.499 (0.416–0.561)	0.518 (0.053) 0.527 (0.407–0.591)	0.002
0.7 mm	0.761 (0.050) 0.765 (0.682–0.870)	0.690 (0.061) 0.681 (0.572–0.767)	0.707 (0.073) 0.722 (0.560–0.807)	0.002
0.8 mm	0.997 (0.066) 1.001 (0.893–1.141)	0.904 (0.078) 0.892 (0.755–1.006)	0.927 (0.094) 0.949 (0.740–1.057)	0.002
0.9 mm	1.267 (0.084) 1.271 (1.133–1.449)	1.148 (0.098) 1.132 (0.966–1.280)	1.178 (0.120) 1.209 (0.949–1.344)	0.001
1.0 mm	1.570 (0.105) 1.574 (1.401–1.795)	1.423 (0.120) 1.403 (1.207–1.588)	1.461 (0.147) 1.504 (1.186–1.667)	0.001
1.1 mm	1.908 (0.128) 1.911 (1.670–2.181)	1.729 (0.143) 1.704 (1.479–1.933)	1.776 (0.178) 1.828 (1.454–2.027)	0.001
1.2 mm	2.281 (0.154) 2.283 (2.027–2.608)	2.067 (0.169) 2.037 (1.785–2.315)	2.124 (0.211) 2.180 (1.749–2.424)	0.001
1.3 mm	2.690 (0.182) 2.690 (2.385–3.075)	2.438 (0.196) 2.401 (2.121–2.733)	2.507 (0.246) 2.567 (2.062–2.862)	0.001
1.4 mm	3.136 (0.214) 3.133 (2.774–3.586)	2.842 (0.225) 2.797 (2.492–3.186)	2.926 (0.283) 2.989 (2.402–3.334)	0.001
1.5 mm	3.620 (0.250) 3.614 (3.196–4.138)	3.280 (0.256) 3.228 (2.911–3.683)	3.379 (0.328) 3.445 (2.765–3.854)	0.001
